# Machine learning to predict end stage kidney disease in chronic kidney disease

**DOI:** 10.1038/s41598-022-12316-z

**Published:** 2022-05-19

**Authors:** Qiong Bai, Chunyan Su, Wen Tang, Yike Li

**Affiliations:** 1 Department of Nephrology, Peking University Third Hospital, 49 North Garden Rd, Haidian District, Beijing, 100191 People’s Republic of China; 2 Department of Otolaryngology-Head and Neck Surgery, Bill Wilkerson Center, Vanderbilt University Medical Center, Nashville, TN USA

**Keywords:** Nephrology, Kidney diseases, Chronic kidney disease, End-stage renal disease

## Abstract

The purpose of this study was to assess the feasibility of machine learning (ML) in predicting the risk of end-stage kidney disease (ESKD) from patients with chronic kidney disease (CKD). Data were obtained from a longitudinal CKD cohort. Predictor variables included patients’ baseline characteristics and routine blood test results. The outcome of interest was the presence or absence of ESKD by the end of 5 years. Missing data were imputed using multiple imputation. Five ML algorithms, including logistic regression, naïve Bayes, random forest, decision tree, and K-nearest neighbors were trained and tested using fivefold cross-validation. The performance of each model was compared to that of the Kidney Failure Risk Equation (KFRE). The dataset contained 748 CKD patients recruited between April 2006 and March 2008, with the follow-up time of 6.3 ± 2.3 years. ESKD was observed in 70 patients (9.4%). Three ML models, including the logistic regression, naïve Bayes and random forest, showed equivalent predictability and greater sensitivity compared to the KFRE. The KFRE had the highest accuracy, specificity, and precision. This study showed the feasibility of ML in evaluating the prognosis of CKD based on easily accessible features. Three ML models with adequate performance and sensitivity scores suggest a potential use for patient screenings. Future studies include external validation and improving the models with additional predictor variables.

## Introduction

Chronic kidney disease (CKD) is a significant healthcare burden that affects billions of individuals worldwide^1,2^ and makes a profound impact on global morbidity and mortality^3–5^. In the United States, approximately 11% of the population or 37 million people suffer from CKD that results in an annual Medicare cost of $84 billion^6^. The prevalence of this disease is estimated at 10.8% in China, affecting about 119.5 million people^7^.

Gradual loss of the kidney function can lead to end stage kidney disease (ESKD) in CKD patients, precipitating the need for kidney replacement therapy (KRT). Timely intervention in those CKD patients who have a high risk of ESKD may not only improve these patients’ quality of life by delaying the disease progression, but also reduce the morbidity, mortality and healthcare costs resulting from KRT^8,9^. Because the disease progression is typically silent^10^, a reliable prediction model for risk of ESKD at the early stage of CKD can be clinically essential. Such a model is expected to facilitate physicians in making personalized treatment decisions for high-risk patients, thereby improving the overall prognosis and reducing the economic burden of this disease.

A few statistical models were developed to predict the likelihood of ESKD based on certain variables, including age, gender, lab results, and most commonly, the estimated glomerular filtration rate (eGFR) and albuminuria^11,12^. Although some of these models demonstrated adequate predictability in patients of a specific race, typically Caucasians^13–15^, literature on their generalizability in other ethnic groups, such as Chinese, remains scarce^13,16^. In addition, models based on non-urine variables, such as patients’ baseline characteristics and routine blood tests, have reportedly yield sufficient performance^17,18^. Therefore, it may be feasible to predict ESKD without urine tests, leading to a simplified model with equivalent reliability.

With the advent of the big data era, new methods became available in developing a predictive model that used to rely on traditional statistics. Machine learning (ML) is a subset of artificial intelligence (AI) that allows the computer to perform a specific task without explicit instructions. When used in predictive modeling, ML algorithm can be trained to capture the underlying patterns of the sample data and make predictions about the new data based on the acquired information^19^. Compared to traditional statistics, ML represents more sophisticated math functions and usually results in better performance in predicting an outcome that is determined by a large set of variables with non-linear, complex interactions^20^. ML has recently been applied in numerous studies and demonstrated high level of performance that surpassed traditional statistics and even humans^20–23^.

This article presents a proof-of-concept study with the major goal to establish ML models for predicting the risk of ESKD on a Chinese CKD dataset. The ML models were trained and tested based on easily obtainable variables, including the baseline characteristics and routine blood tests. Results obtained from this study suggest not only the feasibility of ML models in performing this clinically critical task, but also the potential in facilitating personalized medicine.

## Materials and methods

### Study population

The data used for this retrospective work were obtained from a longitudinal cohort previously enrolled in an observational study^24,25^. The major inclusion criteria for the cohort were adult CKD patients (≥ 18 years old) with stable kidney functions for at least three months prior to recruitment. Patients were excluded if they had one or more of the following situations: (1) history of KRT in any form, including hemodialysis, peritoneal dialysis or kidney transplantation; (2) any other existing condition deemed physically unstable, including life expectancy < 6 months, acute heart failure, and advanced liver disease; (3) any pre-existing malignancy. All patients were recruited from the CKD management clinic of Peking University Third Hospital between April 2006 and March 2008. Written informed consent was obtained from all patients. They were treated according to routine clinical practice determined by the experienced nephrologists and observed until December 31^st^, 2015. Detailed information regarding patient recruitment and management protocol has been described in a previous publication^24^.

### Data acquisition

Patient characteristics included age, gender, education level, marriage status, and insurance status. Medical history comprised history of smoking, history of alcohol consumption, presence of each comorbid condition—diabetes, cardiovascular disease and hypertension. Clinical parameters contained body mass index (BMI), systolic pressure and diastolic pressure. Blood tests consisted of serum creatinine, uric acid, blood urea nitrogen, white blood cell count, hemoglobin, platelets count, alanine aminotransferase (ALT), aspartate aminotransferase (AST), total protein, albumin, alkaline phosphatase (ALP), high-density lipoprotein, low-density lipoprotein, triglycerides, total cholesterol, calcium, phosphorus, potassium, sodium, chloride, and bicarbonate. The estimated glomerular filtration rate and type of primary kidney disease were also used as predictors.

All baseline variables were obtained at the time of subject enrollment. The primary study end point was kidney failure which necessitated the use of any KRT. Subjects with the outcome of kidney failure were labeled as ESKD+, and the rest ESKD−. Patients who died before reaching the study end point or lost to follow up were discarded. Patients who developed ESKD after five years were labeled as ESKD−.

### Data preprocessing

All categorical variables, such as insurance status, education, and primary disease, were encoded using the one-hot approach. Any variable was removed from model development if the missing values were greater than 50%. Missing data were handled using multiple imputation with five times of repetition, leading to five slightly different imputed datasets where each of the missing values was randomly sampled from their predictive distribution based on the observed data. On each imputed set, all models were trained and tested using a fivefold cross validation method. To minimize selection bias, subject assignment to train/test folds was kept consistent across all imputed sets. Data were split in a stratified fashion to ensure the same distribution of the outcome classes (ESKD+ vs. ESKD−) in each subset as the entire set.

### Model development

The model was trained to perform a binary classification task with the goal of generating the probability of ESKD+ based on the given features. Five ML algorithms were employed in this study, including logistic regression, naïve Bayes, random forest, decision tree, and K-nearest neighbors. Grid search was performed to obtain the best hyperparameter combination for each algorithm.

### Assessment of model performance

The performance of a classifiers was measured using accuracy, precision, recall, specificity, F1 score and area under the curve (AUC), as recommended by guidelines for results reporting of clinical prediction models^26^. All classifiers developed in this study were further compared with the Kidney Failure Risk Equation (KFRE), which estimates the 5-year risk of ESKD based on patient’s age, gender, and eGFR^12^. The KFRE is currently the most widely used model in predicting CKD progression to ESKD. The reported outcome of a model represented the average performance of 5 test folds over all imputed sets.

### Statistical analysis

Basic descriptive statistics were applied as deemed appropriate. Results are expressed as frequencies and percentages for categorical variables; the mean ± standard deviation for continuous, normally distributed variables; and the median (interquartile range) for continuous variables that were not normally distributed. Patient characteristics were compared between the original dataset and the imputed sets using one-way analysis of variance (ANOVA). The AUC of each model was measured using the predicted probability. The optimal threshold of a classifier was determined based on the receiver operating characteristic (ROC) curve at the point with minimal distance to the upper left corner. For each ML model, this threshold was obtained during the training process and applied unchangeably to the test set. For the KFRE, the threshold was set at a default value of 0.5. Model development, performance evaluation and data analyses were all performed using Python^27^. The alpha level was set at 0.05.

### Ethical approval

This research was conducted ethically in accordance with the World Medical Association Declaration of Helsinki. The study protocol has been approved by the Peking University Third Hospital Medical Science Research Ethics Committee on human research (No. M2020132).

## Results

### Cohort characteristics

The dataset contained a total of 748 subjects with the follow-up duration of 6.3 ± 2.3 years. The baseline characteristics are summarized in Table 1. Most patients were in stage 2 (24.5%) or 3 (47.1%) CKD at baseline. ESKD was observed in 70 patients (9.4%), all of whom subsequently received KRT, including hemodialysis in 49 patients, peritoneal dialysis in 17 and kidney transplantation in 4.

**Table 1:** Baseline patient characteristics.

Variables	Original data
Age (years)	57.8 ± 17.6
Gender (male/female)	419/329
SBP (mmHg)	129.5 ± 17.8
DBP (mmHg)	77.7 ± 11.1
BMI (kg/m^2^)	24.8 ± 3.7
**Primary disease**	
Primary GN	292 (39.0%)
Diabetes	224 (29.9%)
Hypertension	97 (13.0%)
CIN	64 (8.6%)
Others	18 (2.4%)
Unknown	53 (7.1%)
Creatinine (µmol/L)	130.0 (100.0, 163.0)
Urea (mmol/L)	7.9 (5.6, 10.4)
ALT (U/L)	17.0 (12.0, 24.0)
AST (U/L)	18.0 (15.0, 22.0)
ALP (U/L)	60.0 (50.0, 75.0)
Total protein (g/L)	71.6 ± 8.4
Albumin (g/L)	42.2 ± 5.6
Urine acid (µmol/L)	374.0 (301.0, 459.0)
Calcium (mmol/L)	2.2 ± 0.1
Phosphorous (mmol/L)	1.2 ± 0.2
Ca × P (mg^2^/dL^2^)	33.5 ± 5.6
Blood leukocyte (10^9^/L)	7.1 ± 2.4
Hemoglobin (g/L)	131.0 ± 20.3
Platelet (10^9^/L)	209.8 ± 57.1
eGFR (ml/min/1.73m^2^)	46.1 (32.6, 67.7)
**CKD stage**	
Stage 1	58 (7.8%)
Stage 2	183 (24.5%)
Stage 3	352 (47.1%)
Stage 4	119 (15.9%)
Stage 5	36 (4.8%)
Total cholesterol	5.1 (4.3, 5.9)
Triglyceride	1.8 (1.3, 2.6)
HDL-c	1.3 (1.1, 1.6)
LDL-c	3.0 (2.4, 3.7)
Fasting glucose (mmol/L)	5.4 (4.9, 6.2)
Potassium (mmol/L)	4.3 ± 0.5
Sodium (mmol/L)	140.2 ± 2.8
Chlorine (mmol/L)	106.9 ± 3.7
Bicarbonate (mmol/L)	25.9 ± 3.6
**Medical history**	
Hypertension	558 (74.6%)
Diabetes mellitus	415 (55.5%)
Cardiovascular or cerebrovascular disease	177 (23.7%)
Smoking	91 (12.6%)

*SBP* systolic blood pressure, *DBP* diastolic blood pressure, *GN* glomerulonephritis, *CIN* chronic interstitial nephritis, *BMI* body mass index, *eGFR* estimated glomerular filtration rate, *ALT* alanine aminotransferase, *AST* aspartate transaminase, *ALP* alkaline phosphatase, *CKD* chronic kidney disease, *HDL-c* high density lipoprotein cholesterol, *LDL-c* low density lipoprotein cholesterol, *Ca* × *P* calcium-phosphorus product.

### Model performance

**Figure 1:**
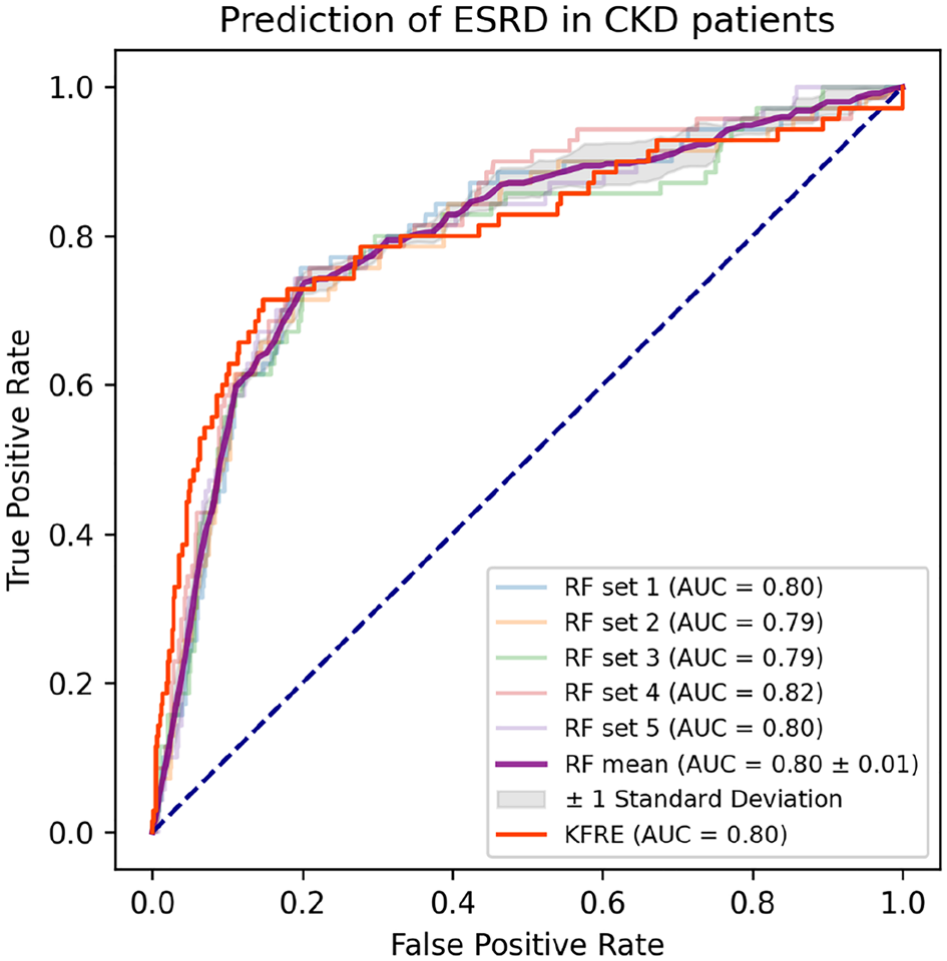
ROC curves of the random forest algorithm and the KFRE model.

Details of the five imputed sets are provided in the supplemental materials. There was no significant difference between the imputed sets and the original dataset in each variable where missing data were replaced by imputed values. The hyperparameter settings for each classifier are displayed in Table 2. The best overall performance, as measured by the AUC score, was achieved by the random forest algorithm (0.81, see Table 3). Nonetheless, this score and its 95% confidence interval had overlap with those of the other three models, including the logistic regression, naïve Bayes, and the KFRE (Fig. 1). Interestingly, the KFRE model that was based on 3 simple variables, demonstrated not only a comparable AUC score but also the highest accuracy, specificity, and precision. At the default threshold, however, the KFRE was one of the least sensitive models (47%).

**Table 2:** Hyperparameters of the algorithms.

Algorithms	Hyperparameters
Logistic regression	penalty = 'l2', class_weight = 'balanced', max_iter = 100000, C = 10, solver = 'liblinear'
Naive Bayes	type = 'multinomial', alpha = 150
Decision tree	criterion = 'gini', splitter = 'best', max_depth = 16, max_features = 15, min_samples_leaf = 5, min_samples_split = 0.0001
Random forest	class_weight = 'balanced', criterion = 'gini', max_depth = 9, max_features = 17, min_samples_leaf = 6, min_samples_split = 30, n_estimators = 32
K-nearest neighbors	weights = 'distance', metric = 'minkowski', n_neighbors = 16, leaf_size = 10

**Table 3:** The performance of all algorithms.

	Accuracy	Sensitivity	Specificity	Precision	F1 Score	AUC
Logistic regression	0.75 (0.72, 0.79)	0.79 (0.73, 0.85)	0.75 (0.71, 0.79)	0.26 (0.24, 0.29)	0.38 (0.36, 0.41)	0.79 (0.77, 0.82)
Naïve Bayes	0.86 (0.85, 0.87)	0.72 (0.68, 0.75)	0.87 (0.86, 0.89)	0.37 (0.35, 0.40)	0.49 (0.46, 0.51)	0.80 (0.77, 0.82)
Random forest	0.82 (0.80, 0.85)	0.76 (0.71, 0.81)	0.83 (0.80, 0.86)	0.34 (0.30, 0.39)	0.46 (0.43, 0.49)	0.81 (0.78, 0.83)
K nearest neighbor	0.84 (0.81, 0.86)	0.60 (0.57, 0.64)	0.86 (0.83, 0.89)	0.35 (0.30, 0.40)	0.43 (0.40, 0.46)	0.73 (0.71, 0.75)
Decision tree	0.84 (0.82, 0.86)	0.44 (0.39, 0.49)	0.89 (0.86, 0.91)	0.33 (0.26, 0.40)	0.35 (0.32, 0.39)	0.66 (0.63, 0.68)
KFRE	0.90 (0.90, 0.91)	0.47 (0.42, 0.52)	0.95 (0.94, 0.96)	0.50 (0.45, 0.55)	0.48 (0.43, 0.52)	0.80 (0.78, 0.83)

All outcomes are expressed as mean and (95% confidence interval).

*KFRE* kidney failure risk equation, *AUC* area under the curve.

## Discussion

With extensive utilization of electronic health record and recent progress in ML research, AI is expanding its impact on healthcare and has gradually changed the way clinicians pursue for problem-solving^28^. Instead of adopting a theory-driven strategy that requires a preformed hypothesis from prior knowledge, training an ML model typically follows a data-driven approach that allows the model to learn from experience alone. Specifically, the model improves its performance iteratively on a training set by comparing the predictions to the ground truths and adjusting model parameters so as to minimize the distance between the predictions and the truths. In nephrology, ML has demonstrated promising performances in predicting acute kidney injury or time to allograft loss from clinical features^29,30^, recognizing specific patterns in pathology slides^31,32^, choosing an optimal dialysis prescription^33^, or mining text in the electronic health record to find specific cases^34,35^. Additionally, a few recent studies were performed to predict the progression of CKD using ML methods. These models were developed to estimate the risk of short-term mortality following dialysis^36^, calculate the future eGFR values^37^, or assess the 24-h urinary protein levels^18^. To our best knowledge, there hasn’t been any attempt to apply ML methods to predict the occurrence of ESKD in CKD patients.

In the present study, a prediction model for ESKD in CKD patients was explored using ML techniques. Most classifiers demonstrated adequate performance based on easily accessible patient information that is convenient for clinical translation. In general, three ML models, including the logistic regression, naïve Bayes and random forest, showed non-inferior performance to the KFRE in this study. These findings imply ML as a feasible approach for predicting disease progression in CKD, which could potentially guide physicians in establishing personalized treatment plans for this condition at an early stage. These ML models with higher sensitivity scores may also be practically favored in patient screening over the KFRE.

To our best understanding, this study was also the first to validate the KFRE in CKD patients of Mainland China. The KFRE was initially developed and validated using North American patients with CKD stage 3–5^12^. There were seven KFRE models that consisted of different combinations of predictor variables. The most commonly used KFRE included a 4-variable model (age, gender, eGFR and urine ACR) or an 8-variable model (age, gender, eGFR, urine ACR, serum calcium, phosphorous, bicarbonate, and albumin). Besides, there was a 3-variable model (age, gender, and eGFR) that required no urine ACR and still showed comparable performance to the other models in the original article. Despite its favorable performance in prediction for ESKD in patients of Western countries^14,15,38,39^, the generalizability of KFRE in Asian population remained arguable following the suboptimal results revealed by some recent papers^13,40,41^. In the current study, the KFRE was validated in a Chinese cohort with CKD stage 1–5 and showed an AUC of 0.80. This result indicated the KFRE was adequately applicable to the Chinese CKD patients and even earlier disease stages. In particular, the high specificity score (0.95) may favor the use of this equation in ruling in patients who require close monitoring of disease progression. On the other hand, a low sensitivity (0.47) at the default threshold may suggest it may be less desirable than the other models for ruling out patients.

Urine test is a critical diagnostic approach for CKD. The level of albuminuria (i.e. ACR) has also been regarded as a major predictor for disease progression and therefore used by most prognostic models. However, quantitative testing for albuminuria is not always available in China especially in rural areas, which precludes clinicians from using most urine-based models for screening patients. In this regard, several simplified models were developed to predict CKD progression without the need of albuminuria. These models were based on patient characteristics (e.g. age, gender, BMI, comorbidity) and/or blood work (e.g. creatinine/eGFR, BUN), and still able to achieve an AUC of 0.87–0.89^12,18^ or a sensitivity of 0.88^37^. Such performance was largely consistent with the findings of this study and comparable or even superior to some models incorporating urine tests^16,42^. Altogether, it suggested a reliable prediction for CKD progression may be obtained from routine clinical variables without urine measures. These models are expected to provide a more convenient screening tool for CKD patients in developing regions.

Missing data are such a common problem in ML research that they can potentially lead to a biased model and undermine the validity of study outcomes. Traditional methods to handle missing data include complete case analysis, missing indicator, single value imputation, sensitivity analyses, and model-based methods (e.g. mixed models or generalized estimating equations)^43–45^. In most scenarios, complete case analysis and single value imputation are favored by researchers primarily due to the ease of implementation^45–47^. However, these methods may be associated with significant drawbacks. For example, by excluding samples with missing data from analyses, complete case analysis can result in reduction of model power, overestimation of benefit and underestimation of harm^43,46^; Single value imputation replaces the missing data by a single value—typically the mean or mode of the complete cases, thereby increasing the homogeneity of data and overestimating the precision^43,48^. In this regard, multiple imputation solves these problems by generating several different plausible imputed datasets, which account for the uncertainty about the missing data and provide unbiased estimates of the true effect^49,50^. It is deemed effective regardless of the pattern of missingness^43,51^. Multiple imputation is now widely recognized as the standard method to deal with missing data in many areas of research^43,45^. In the current study, a 5-set multiple imputation method was employed to obtain reasonable variability of the imputed data. The performance of each model was analyzed on each imputed set and pooled for the final result. These procedures ensured that the model bias resulting from missing data was minimized. In the future, multiple imputation is expected to become a routine method for missing data handling in ML research, as the extra amount of computation associated with multiple imputation over those traditional methods can simply be fulfilled by the high level of computational power required by ML.

Although ML has been shown to outperform traditional statistics in a variety of tasks by virtue of the model complexity, some studies demonstrated no gain or even declination of performance compared to traditional regression methods^52,53^. In this study, the simple logistic regression model also yielded a comparable or even superior predictability for ESKD to other ML algorithms. The most likely explanation is that the current dataset only had a small sample size and limited numbers of predictor variables, and the ESKD+ cases were relatively rare. The lack of big data and imbalanced class distribution may have negative impact on the performance of complex ML algorithms, as they are typically data hungry^54^. On the other hand, this finding could imply simple interactions among the predictor variables. In other words, the risk of ESKD may be largely influenced by only a limited number of factors in an uncomplicated fashion, which is consistent with some previous findings^12,18,55^. The fact that the 3-variable KFRE, which is also a regression model, yielded equivalent outcomes to the best ML models in this study may further support this implication. It is therefore indicated that traditional regression models may continue to play a key role in disease risk prediction, especially when a small sample size, limited predictor variables, or an imbalanced dataset is encountered. The fact that some of the complex ML models are subject to the risk of overfitting and the lack of interpretability further favors the use of simple regression models, which can be translated to explainable equations.

Several limitations should be noted. First, this cohort consisted of less than 1000 subjects and ESKD only occurred in a small portion of them, both of which might have affected model performance as discussed earlier. Second, although this study aimed to assess the feasibility of a prediction model for ESKD without any urine variables, this was partially due to the lack of quantitative urine tests at our institute when this cohort was established. As spot urine tests become increasingly popular, urine features such as ACR will be as accessible and convenient as other lab tests. They are expected to play a critical role in more predictive models. Third, the KFRE was previously established on stages 3–5 CKD patients while the current cohort contained stages 1–5. This discrepancy may have affected the KFRE performance. Forth, the generalizability of this model has not been tested on any external data due to the lack of such resource in this early feasibility study. Therefore, additional efforts are required to improve and validate this model before any clinical translation. Finally, although a simple model without urine variables is feasible and convenient, model predictability may benefit from a greater variety of clinical features, such as urine tests, imaging, or biopsy. Future works should include training ML models with additional features using a large dataset, and validating them on external patients.

In conclusion, this study showed the feasibility of ML in evaluating the prognosis of CKD based on easily accessible features. Logistic regression, naïve Bayes and random forest demonstrated comparable predictability to the KFRE in this study. These ML models also had greater sensitivity scores that were potentially advantageous for patient screenings. Future studies include performing external validation and improving the model with additional predictor variables.

## Supplementary Information

Supplementary Information.
