# Supplementary material for: Machine learning to predict end stage kidney disease in chronic kidney disease
Source: Sci Rep. 2022 May 19;12:8377. doi: 10.1038/s41598-022-12316-z (PMC9120106; doi:10.1038/s41598-022-12316-z)
Supplement: Supplementary file 1 — Supplementary Information. [file 41598_2022_12316_MOESM1_ESM.docx]

Table. Clinical parameters of all patients at baseline

| Variables | Original data | Missing data  N (%) | Imputation 1 | Imputation 2 | Imputation 3 | Imputation 4 | Imputation 5 | *P* value |
| --- | --- | --- | --- | --- | --- | --- | --- | --- |
| Age (years)  SBP (mmHg)  DBP (mmHg)  BMI (kg/m^2^)  Primary disease  Primary GN  Diabetes  Hypertension  CIN  Others  Unknown  Creatinine (µmol/L)  Urea (mmol/L)  Total protein (g/L)  Albumin (g/L)  ALT (U/L)  AST (U/L)  ALP (U/L)  Urine acid (µmol/L)  Calcium (mmol/L)  Phosphorous (mmol/L)  Ca×P (mg^2^/dL^2^)  Blood leukocyte (10^9^/L)  Hemoglobin (g/L)  Platelet (10^9^/L) | 57.8±17.6  129.6±17.8  77.7±11.1  24.8±3.7  292 (39.0%)  224 (29.9%)  97 (13.0%)  64 (8.6%)  18 (2.4%)  53 (7.1%)  130.0 (100.0, 163.0)  7.9 (5.6 10.4)  71.6±8.4  42.2±5.5  17.0 (12.0, 24.0)  18.0 (15.0, 22.0)  60.0 (50.0, 75.0)  374.0 (301.0, 459.0)  2.2±0.1  1.2±0.2  33.5±5.6  7.1±2.4  131.0±20.3  209.8±57.1 | 0  13 (1.7%)  13 (1.7%)  10 (1.3%)  0  0  17 (2.3%)  271 (36.2%)  249 (33.3%)  213 (28.5%)  227 (30.3%)  315 (42.1%)  45 (6.0%)  92 (12.3%)  104 (13.9%)  104 (13.9%)  291 (38.9%)  290 (38.8%)  298 (39.8%) | 129.4±17.7  77.6±11.1  24.8±3.7  7.9 (5.7, 10.5)  71.6±8.7  42.2±5.5  19.0 (13.0, 27.7)  19.0 (15.5, 23.7)  61.0 (50.0, 77.0)  374.5 (302.0, 462.7)  2.2±0.2  1.2±0.2  33.5±5.4  7.2±2.4  132.8±20.3  210.3±58.4 | 129.5±17.8  77.7±11.1  24.7±3.7  7.9 (5.6, 10.4)  71.6±8.7  42.3±5.6  18.0 (12.9, 26.0)  18.8 (15.0 23.0)  61.0 (50.0, 76.4)  375.5 (304.0, 465.7)  2.2±0.2  1.2±0.2  33.4±5.4  7.3±2.4  132.7±20.8  208.3±57.8 | 129.5±17.9  77.6±11.1  24.7±3.7  7.9 (5.6, 10.4)  72.1±8.4  42.3±5.5  19.0 (13.0, 28.6)  19.0 (15.0, 24.0)  62.0 (50.0, 77.3)  375.0 (301.7, 462.2)  2.2±0.1  1.2±0.2  33.6±5.4  7.3±2.4  132.7±20.0  210.8±58.7 | 129.5±17.8  77.7±11.2  24.7±3.7  7.9 (5.7, 10.5)  72.1±8.6  42.4±5.6  19.0 (13.0, 27.0)  19.0 (15.0, 23.1)  61.0 (50.0, 76.0)  374.0 (302.0, 459.4.0)  2.2±0.2  1.2±0.2  33.6±5.4  7.2±2.3  133.3±19.9  208.3±57.5 | 129.5±17.9  77.7±11.1  24.8±3.7  7.9 (5.7, 10.5)  71.7±8.4  42.3±5.5  19.0 (13.0, 28.0)  19.0 (15.0, 24.0)  61.7 (50.0, 76.4)  374.0 (304.0, 461.1)  2.2±0.2  1.2±0.2  33.5±5.4  7.3±2.4  132.9±20.1  209.2±56.1 | 1.000  1.000  1.000  0.999  0.682  0.975  0.412  0.413  0.584  0.995  0.955  0.998  0.992  0.678  0.560  0.950 |
| eGFR(ml/min/1.73m^2^)  CKD stage  Stage 1  Stage 2  Stage 3  Stage 4  Stage 5 | 46.1 (32.6, 67.7)  58 (7.8%)  183 (24.5%)  352 (47.1%)  119 (15.9%)  36 (4.8%) | 0  0 |  |  |  |  |  |  |
| Total cholesterol  Triglyceride  HDL-c  LDL-c  Potassium (mmol/L)  Sodium (mmol/L)  Chlorine (mmol/L)  Bicarbonate (mmol/L)  Medical History  Hypertension  Diabetes mellitus  Cardiovascular or  cerebrovascular disease  Smoking | 5.1 (4.3, 5.9)  1.8 (1.3, 2.6)  1.3 (1.1, 1.6)  3.0 (2.4, 3.7)  4.3±0.5  140.2±2.8  106.9±3.7  25.9±3.6  558 (74.6%)  415 (55.5%)  177 (23.7%)  91 (12.6%) | 208 (27.8%)  209 (27.9%)  223 (29.8%)  228 (30.5%)  263 (35.2%)  297 (39.7%)  306 (40.9%)  318 (42.5%)  0  26 (3.5%) | 5.1 (4.3, 6.0)  1.9 (1.3, 2.7)  1.3 (1.1, 1.7)  3.0 (2.3, 3.7)  4.3±0.5  140.4±2.9  106.9±3.7  26.2±3.5  94 (12.6%) | 5.0 (4.3, 5.9)  1.9 (1.3, 2.7)  1.3 (1.1, 1.6)  3.0 (2.3, 3.7)  4.3±0.5  140.1±2.9  106.7±3.7  26.2±3.6  93 (12.4%) | 5.1 (4.3, 6.0)  1.9 (1.3, 2.8)  1.3 (1.1, 1.6)  3.0 (2.4, 3.9)  4.3±0.5  140.2±2.9  106.9±3.8  26.0±3.5  94 (12.6%) | 5.1 (4.3, 6.0)  1.9 (1.3, 2.8)  1.3 (1.1, 1.6)  3.0 (2.4, 3.8)  4.3±0.5  140.1±2.8  106.6±3.7  26.2±3.6  94 (12.6%) | 5.1 (4.3, 6.0)  1.9 (1.3, 2.7)  1.3 (1.1, 1.6)  3.0 (2.4, 3.8)  4.3±0.5  140.3±2.9  106.9±3.7  26.1±3.6  94 (12.6%) | 0.575  0.614  0.729  0.967  0.728  0.204  0.815  0.870  1.000 |

Abbreviations: SBP, systolic blood pressure; DBP, diastolic blood pressure; GN, glomerulonephritis; CIN, chronic interstitial nephritis; BMI, body mass index; eGFR, estimated glomerular filtration rate; ALT, alanine aminotransferase; AST, aspartate transaminase; ALP, alkaline phosphatase; CKD, chronic kidney disease; HDL-c, high density lipoprotein cholesterol; LDL-c, low density lipoprotein cholesterol; Ca×P, calcium-phosphorus product; Total CO_2_, total carbon dioxide.
